# Culturally and linguistically diverse patients in an Italian memory clinic: lessons learned from the ImmiDem project

**DOI:** 10.1007/s10072-026-08860-5

**Published:** 2026-02-24

**Authors:** I. Cova, G. Maestri, A. Nicotra, A. Forgione, L. Maggiore, A. Acampora, M. Canevelli, N. Vanacore, L. Pantoni, S. Pomati

**Affiliations:** 1 Neurology Unit, Luigi Sacco University Hospital, Milan, Italy; 2 Department of Epidemiology, Regional Health Service, Lazio Region, Rome, Italy; 3 Department of Human Neuroscience, “Sapienza” University, Rome, Italy; 4 National Center for Disease Prevention and Health Promotion, Italian National Institute of Health, Rome, Italy; 5 Department of Biomedical and Clinical Sciences, University of Milan, Milan, Italy; 6 Department of Neurorehabilitation Sciences, Casa Di Cura Igea, Milan, Italy; 7 Istituti Clinici Scientifici Maugeri IRCCS, Pavia, Italy

**Keywords:** Awareness, Cognitive assessment, Cross-cultural, Epidemiology, Diversity, Inequalities

## Abstract

**Background:**

Increasing migration and population ageing pose growing challenges for dementia diagnosis in culturally and linguistically diverse (CALD) populations. This study evaluated changes in diagnostic practices in an Italian Centre for Cognitive Disorders and Dementia following implementation of the ImmiDem project and culturally adapted cognitive tools.

**Methods:**

A retrospective observational study was conducted including all CALD patients assessed between 2016 and 2024. Collected data included demographic and linguistic characteristics, country of origin, type of cognitive assessment, and use of standard versus cross-cultural tools. Diagnostic practices were compared between T1 (2016–2020) and T2 (2021–2024), corresponding to ImmiDem implementation.

**Results:**

A total of 163 CALD patients (4.2% of referrals) were identified, with a marked increase in T2 (+ 180%). Mean age was 65.2 ± 13.8 years; 35.2% had low education. Patients originated from 46 countries and spoke 26 languages, with no significant demographic differences between T1 and T2. Cognitive screening was performed in 81.6% and extensive testing in 33.1%. Use of cultural mediators and cross-cultural tools rose significantly in T2. Importantly, diagnoses in T2 more frequently involved milder cognitive impairment, likely reflecting earlier referral and the increasing use of more sensitive cross-cultural cognitive assessment tools.

The Rowland Universal Dementia Assessment Scale (RUDAS) showed weaker correlations with age and education than the Mini-Mental State Examination (MMSE) and the Montreal Cognitive Assessment (MoCA), with generally good concordance across tools.

**Conclusions:**

Referrals of CALD patients increased substantially, with earlier detection of cognitive impairment and wider use of culturally appropriate assessment. Persistent gaps in validated tools and cultural competence highlight the need for further multicentre prospective studies to support equitable diagnostic practices.

## Introduction

Dementia is recognized as a growing public health challenge globally, and is gaining increasing relevance among migrants and ethnically diverse populations [1]. Migrants may face a higher risk of delayed diagnosis and suboptimal care due to linguistic, cultural, and socioeconomic barriers that can compromise access to appropriate services and the validity of standard cognitive assessments [2]. In this context, ensuring equity in dementia diagnosis and care constitutes both a clinical and a public health priority [3]. Recent international frameworks, such as the Global Evidence on Health and Migration (GEHM) coordinated by the World Health Organization (WHO) [4], have underscored the need to strengthen health systems’ preparedness to meet the complex needs of migrant populations, including those affected by cognitive decline.

An early study conducted in 2011 by the European Alzheimer's Disease Consortium (EADC) [5], which involved 15 dementia centres across Europe, found that although patients from ethnic minorities were seen regularly, cognitive assessments were primarily conducted using tools validated only in Western populations. These assessments often depended on family members for interpretation. More than half of the centres reported challenges in diagnostic evaluation, mainly due to language barriers and a lack of culturally appropriate assessment instruments. Overall, the study identified a poor preparedness in providing culturally sensitive dementia care. A 2024 follow-up study [6] concluded that, despite some improvements, diagnostic difficulties persisted, underscoring the continuing need for effective cross-cultural communication and assessment practices.

In Italy, previous studies [7, 8] have shown that migrants living with cognitive disorders constitute a highly heterogeneous patient population, with wide variations in language, country of origin, and diagnostic approaches in terms of neuropsychological evaluation. A nationwide survey [9] conducted as part of the ImmiDem project, a research initiative funded by the Italian Ministry of Health to investigate dementia in migrants and ethnic minorities, further confirmed this variability. The study provided unique insights into the scale and characteristics of this emerging phenomenon, documenting regional disparities and inequalities in dementia care for migrants across Italy. Notably, it also highlighted the limited preparedness of the Italian healthcare system and of healthcare professionals in addressing the needs of culturally and linguistically diverse (CALD) patients.

Similar challenges have been documented in other European countries, including Norway [10], Germany [11], and Scotland [12], where national surveys similarly identified significant gaps in culturally competent dementia care. Recent reviews [13, 14] and a position paper [2] further emphasized the ongoing urgency of developing effective strategies for assessing and caring for CALD patients with cognitive impairment.

Building on these premises, several initiatives implemented within the ImmiDem project have aimed to improve clinical practice and service preparedness. These included: i) the dissemination of information materials on dementia and cognitive disturbances in CALD patients via a dedicated website (www.immidem.it); ii) the translation and validation of cross-cultural cognitive and neuropsychological assessment tools (respectively The European cross-cultural neuropsychological test battery [15] and the Brief Assessment of Impaired Cognition (BASIC) and BASIC Questionnaire [16]); and iii) the training of healthcare professionals on culturally sensitive assessment approaches to dementia. Such activities have sought to increase awareness and promote the integration of equitable diagnostic practices across Italian outpatient memory clinics (i.e., Centres for Cognitive Disorders and Dementia (CCDDs)).

This study aimed to evaluate the changes observed in the clinical practice of a tertiary CCDD following the initiatives of the ImmiDem project and the introduction of novel assessment tools more suitable for CALD populations. We sought to evaluate how increased awareness and information dissemination have impacted patient care, and to identify any remaining gaps to guide future improvements in service delivery to inform subsequent research efforts.

## Methods

### Study design and participants

A retrospective observational study was conducted in accordance with the Strengthening the Reporting of Observational Studies in Epidemiology (STROBE) guidelines [17]. All CALD patients consecutively referred to the CCDD of Sacco University Hospital, Milan, between January 2016 and December 2024 were included in this analysis. CALD status was assigned to subjects born abroad whose primary language was different from Italian.

### Data collection

Sociodemographic and clinical data were extracted from electronic medical records and included age, sex, native language, linguistic proficiency, educational level, and country of origin.

Educational attainment was categorized according to the International Standard Classification of Education (ISCED 2011) [18]. Individuals with no formal education up to lower secondary education were classified as having low education, while those with upper secondary, post-secondary, or tertiary education (including doctoral degrees) were classified as having high education.

Countries of origin were grouped following the WHO regional classification—Africa (AFRO), Americas (AMRO), Eastern Mediterranean (EMRO), Europe (EURO), South-East Asia (SEARO), and Western Pacific (WPRO)—and also divided according to the current World Bank Group (WBG) income classification (High-Income [HI], Upper-Middle [UM], Lower-Middle [LM], and Low-Income [LI]). For analytical purposes, LM and LI countries were combined into a single category (WBG-2 classification: HI, UM, and LI–LM). Venezuela, currently unclassified by the WBG, was assigned to the LI–LM group.

### Temporal grouping

The sample was analysed over two time periods: i) T1 (2016–2020), before the launch of the ImmiDem project (2020); and ii) T2 (2021–2024), after the start of ImmiDem. Comparisons were made between these two periods.

### Neuropsychological assessment and cognitive diagnosis

Information was collected regarding the cognitive assessment pathway, distinguishing between screening evaluations (SCR) and extensive neuropsychological assessments (NPS), the specific neuropsychological instruments administered, the presence or role of any intermediary figures involved in test delivery, and the final diagnostic classification. SCR was performed using one or two different tests among the Mini-Mental State Examination (MMSE) [19], the Montreal Cognitive Assessment (MoCA) [20], and the Rowland Universal Dementia Assessment Scale (RUDAS) [21]. NPS was conducted using either standard cognitive tests (SCT) or cross-cultural cognitive tests (CCT) (Table 1). MMSE, MOCA and SCT have been also scored with Equivalent Scores, as defined by Capitani and Laiacona [22], which consist in a non-parametric, norm-based scoring system that transforms raw test scores into ordinal categories (ranging from 0 to 4), adjusted for age and education, where lower values indicate poorer performance; specifically, an Equivalent Score of 0 indicates a pathological performance, a score of 1 a borderline performance, and scores from 2 to 4 performances within the normal range, with increasing levels of adequacy. Diagnostic categories were defined according to current international criteria: no current cognitive complaints (NON), subjective cognitive decline (SCD) [23], mild cognitive impairment (MCI) [24], and dementia (DEM) [25]).

**Table 1:** Standard and cross-cultural neuropsychological tests

Cognitive domain	Standard neuropsychological test	Cross-cultural neuropsychological test
General cognition	MiniMental State Examination Montreal Cognitive Assessment	Rowland Universal Dementia Assessment Scale
Multidomain battery		European Cross-cultural Neuropsychological Test Battery Multicultural Cognitive Examination
Memory	Free and cued selective reminding test Rey figure test—delayed recall	
Attention and executive functions	Symbol digit modalities test Trail making test (TMT, part A and B) Color word Stroop test Attentional matrices Frontal assessment battery	
Language	Phonemic and semantic verbal fluency tasks Picture naming test Screening for aphasia in neurodegeneration	Naming assessment in multicultural europe Bilingual Aphasia Test Switching verbal fluency test
Visuospatial functions	Rey figure test—copy Clock drawing test Apple test	
Other scales/questionnaires	Clinical Dementia Rating Scale Activity of Daily Living Instrumental Activity of Daily Living Neuropsychiatric Inventory Geriatric Depression Scale	Clinical Dementia Rating Scale Activity of Daily Living Instrumental Activity of Daily Living Neuropsychiatric Inventory Brief acculturation scale for hispanics – english

### Comparison with previous Italian studies on CALD patients

Data collected at our CCDD between 2016 and 2024 were compared with findings from the two previously published Italian studies addressing similar populations and settings: Canevelli et al. [7] and Cova et al. [8]. The comparison focused on the sociodemographic and clinical characteristics of CALD patients referred to the centres, as well as on the diagnostic approaches adopted in each study.

### Ethical considerations

The study protocol was approved by the local Ethics Committee of the Sacco University Hospital, Milan. All procedures were conducted in accordance with the principles of the Declaration of Helsinki.

### Statistical analysis

Continuous variables were expressed as mean ± standard deviation (SD). Categorical variables were summarized as absolute frequencies and percentages.

Group comparisons between T1 and T2, sex, educational level, and linguistic proficiency were performed using χ^2^ tests or Fisher’s exact tests for categorical variables; Student’s t-tests or Mann–Whitney U tests were used for continuous variables, depending on their distribution. Correlations among continuous measures (e.g., between test scores and demographic variables) were examined using Pearson’s r coefficients. Agreement between cognitive tests was evaluated with correlation analyses and percentage concordance, and further explored through cross-tabulation of diagnostic classifications.

A two-tailed *p*-value < 0.05 was considered statistically significant.

All data were analysed using IBM SPSS Statistics version 29.0 (IBM Corp., Armonk, NY, USA).

## Results

Table 2 reports a comparison of CALD patient characteristics across time periods and with previous Italian studies.

**Table 2:** Comparison of CALD patient characteristics across time periods and previous Italian studies

	Overall (*n = *163)	T1: 2016–2020 (*n = *35)	T2: 2021–2024 (*n = *128)	Stat	Canevelli (*n = *42)		Cova (*n = *61)
ALL	3843	1676	2167		2851		4701
CALD/all (%)	4.2%	2.1%	5.9%	χ^2^ =.743	1.5%		3.1%
					HIC (*n = *28)	LMIC (*n = *14)	
Age, years	65.2 ± 13.8	65.1 ± 13.3	65.3 ± 14.1	t = -.07	72.9 ± 10.6	70.2 ± 12.4	67.5 ± 14.2
Education, years^§^	11.3 ± 5.2	10.3 ± 4.6	11.5 ± 5.3	t = −1.09	9.3 ± 5.0	10.3 ± 4.9	11.0 ± 5.2
Education < ISCED3 (%)	57 (35.2)	12 (34.3)	38 (32.5)	χ^2^ =.140	-		-
Sex (F): n. (%)	107 (66.6)	22 (62.9)	85 (63.9)	χ^2^ =.154	62.2%	50%	38 (62.3%)
Knowledge of Italian (%) any	130 (79.8)	24 (68.6)	104 (81.3)	χ^2^ =.826	100%		93.4%
WBG-2: LM-LI (%)	55 (33.7)	11 (31.4)	44 (34.4)	χ^2^ =.743	33.3%		-
SCR, n (%)	133 (81.6)	28 (80.0)	105 (82.0)	χ^2^ =.076	98%		65.5%
NPS, n (%)	59 (36.2)	11 (31.4)	48 (37.5)	χ^2^ =.439	-		-
MMSE, n (%)	64 (39.3)	17 (48.6)	47 (36.7)	χ^2^ =.877	98%		65.5%
MoCA, n (%)	58 (35.6)	6 (17.1)	52 (39.1)	χ^2^ = 6.61 *	-		-
RUDAS, n (%)	42 (25.8)	1 (2.9)	41 (30.8)	χ^2^ = 12.2 **	-		-
MMSE score	23.4 ± 6.3	23.0 ± 6.0	23.5 ± 6.4	t = -.31	22.7 ± 7.0	20.0 ± 10.0	23.4 ± 6.2
MoCA score	19.7 ± 5.7	21.0 ± 4.4	19.5 ± 5.8	t =.60	-		-
RUDAS score	21.5 ± 5.8	23.0 ± 0.0	20.5 ± 5.8	t =.43	-		-
Cognitive profile:				χ^2^ = 4.03			
- NON, n (%)	15 (9.2)	1 (2.9)	14 (10.9)		-		19.7%
- SCD, n (%)	29 (17.8)	4 (11.4)	25 (19.5)		-		
- MCI, n (%)	57 (35)	15 (42.9)	42 (32.8)		50.2%	58.2%	16.4%
- DEM, n (%)	62 (38)	15 (42.9)	47 (36.7)				52.5%

^*^ <.05; ** <.001

^§^available for 25 patients in T1 and 117 patients in T2

**Legend:** *CALD* Culturally and Linguistically Diverse; *DEM* Dementia; *MCI* Mild Cognitive Impairment; *HIC* High Income Country; *LMIC* Low-Middle Income Country; *MMSE* Mini-Mental State Examination; *MoCA* Montreal Cognitive Assessment; *NON* no current cognitive complaints; *NPS* extensive neuropsychological assessment; *RUDAS* Rowland Universal Dementia Assessment Scale; *SCD* Subjective cognitive impairment; *SCR* Screening; *WBG* World Bank Group

From 2016 to 2024, 163 CALD patients, of whom 107 (66.6%) were women, were consecutively evaluated, with a mean age of 65.2 ± 13.8 years and a mean education level of 11.3 ± 5.2 years (6.3% were illiterate, and 35.2% had low educational attainment). Most patients were evaluated in the T2 period (2021–2024), with 128 (78.5%) patients assessed compared to 35 (21.5%) in T1 (2016–2020). Overall, CALD patients accounted for 4.2% of all 3,843 individuals evaluated over the study period, with prevalence increasing from 2.1% in T1 to 5.9% in T2.

The majority of patients (79.8%) had some knowledge of the Italian language, with higher language proficiency observed among those with a higher level of education (93.5% in the high education group vs. 66% in the low education group, χ^2^ = 18, *p* < 0.001). No significant differences in level of education were found between male and female patients. Furthermore, no significant differences in age, education, and sex were observed between the T1 and T2 time periods.

**Fig. 1:**
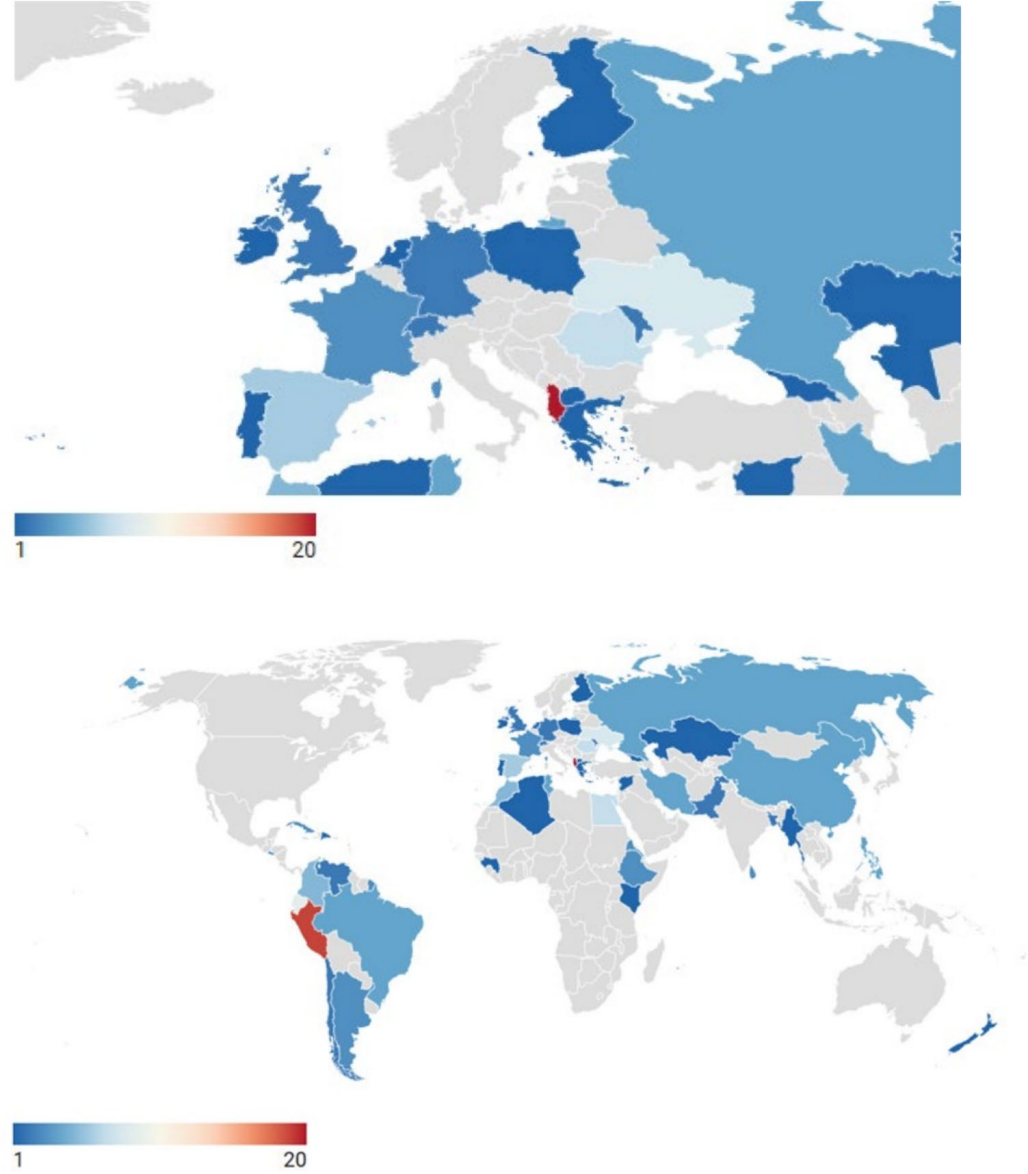
Number of cognitive assessments per country of origin (2016–2024)

Patients originated from 46 different countries, with the largest groups coming from Albania (*n = *20), Peru (*n = *19), Ecuador (*n = *9), and Ukraine (*n = *8) (Fig. 1). Most patients came from the EURO region (*n = *65, 39.9%). When grouped by WBG classification, UM and LI-LM countries were more represented than HI countries, with 78 (47.9%), 55 (33.7%), and 30 (18.4%) patients, respectively. No significant differences were found in the distribution of WBG-2 countries between T1 and T2.

A total of 26 different native languages were identified among the patients, with Spanish being the most common (*n = *52), followed by Albanian (*n = *20) and Arabic (*n = *16).

Regarding clinical presentation, 15 patients (9.2%) had no current cognitive complaints (NON), 29 (17.8%) had a subjective cognitive impairment (SCD), 57 (35%) were diagnosed with mild cognitive impairment (MCI), and 62 (38%) met criteria for dementia (DEM). No significant differences were observed in the distribution of cognitive impairment levels by region, WBG-2, or time frame, nor in the interaction between region/WBG-2 with respect to time frame (T1 vs. T2). Dementia was diagnosed more frequently in subjects without knowledge of the Italian language (NON: 1/13, SCD: 0/26, MCI: 6/51, DEM: 26/36; χ^2^ = 30.1, *p* < 0.001).

SCR was performed in 133 subjects (81.6%)—28 out of 35 in T1 and 105 out of 128 in T2—with no significant difference between periods. NPS was administered to 54 individuals (33.1%)—31.4% in T1, 37.5% in T2—again with no significant difference. SCR was conducted in Italian for 96 patients (20 in T1, 76 in T2); in other cases, the assessment was performed in the patient’s native language, either with an informal interpreter/cultural mediator (typically a family member, occasionally a healthcare professional) in 18 cases (8 in T1, 10 in T2), or with a professional cultural mediator in 19 cases (none in T1, all 19 in T2).

For NPS, 28 assessments were done in Italian (10 in T1, 18 in T2), 4 with an informal mediator (1 in T1, 3 in T2), and 27 with a professional mediator (all in T2). In particular, even among patients with some knowledge of Italian, in approximately half of the cases (24 out of 52; 46.2%) a mediator (formal or informal) was involved, demonstrating the different approaches to evaluation adopted over time.

For individuals who underwent neither SCR nor NPS, 4 had no current cognitive complaints, 6 had SCD (all with some knowledge of Italian), 2 had MCI, and 18 had dementia. In these instances, a presumptive diagnosis was based on thorough clinical history, unstructured objective evaluation, and laboratory or neuroimaging findings.

Screening was performed using MMSE (64 patients, 39.3%; 17 in T1, 47 in T2), MoCA (58 patients, 33.1%; 6 in T1, 48 in T2), or RUDAS (42 patients, 25.8%; 1 in T1, 41 in T2).

Among 28 patients, both MMSE (including MoCA-derived scores [25] and RUDAS were available, with a strong correlation observed between the scores (*r* = 0.764, *p* < 0.001). In 16 patients, both MoCA and RUDAS were available, demonstrating a moderate correlation (*r* = 0.524, p = 0.037).

When comparing classifications between RUDAS and MMSE, all cases with RUDAS < 23 also had an MMSE-equivalent score < 1 [26]. Conversely, in 5 cases, the RUDAS score was > 23 while the MMSE-equivalent was < 1 (raw scores between 22 and 25). Overall agreement between the two tests was 21/26 cases (80.8%). In discordant cases, patients tended to be younger (mean age 63.8 ± 12.4 vs. 69.8 ± 10.1, NS), and their primary language was Spanish (*n = *2), Russian (*n = *2), or Ukrainian (*n = *1); education was slightly higher among discordant cases (agree: 10/11 vs. disagree: 0/5, χ^2^ = 3.87, p = 0.049), with no significant differences in sex or Italian language proficiency. Both MMSE and MoCA scores were positively correlated with education (*r* = 0.357, *p* < 0.001 and *r* = 0.470, *p* < 0.001, respectively) and negatively with age (*r* = −0.353, *p* < 0.001 and *r* = −0.269, p = 0.024), while RUDAS scores showed no significant association with demographic variables.

Cross-cultural tests (CCT) were available for 27 patients (all in the T2 period), standard tests (SCT) were administered to 50 patients, while both types of tests had been employed in 27 cases (meaning every patient assessed with a CCT also underwent at least one SCT).

Among CCT, the most frequently administered were subtests from the Cross-Cultural Neuropsychological Test Battery (CNTB) [15], including the Recall of Pictures Test (RPT, *n = *25), Semi-Complex Figure Test (SCFT, *n = *20), Clock Reading Test (CRT, *n = *18), and Enhanced Cued Recall (ECR, *n = *13). Subtests from the Bilingual Aphasia Test (BAT) [27] assessing verbal fluency, comprehension, and naming were also used (*n = *17). Other CCTs, such as the Naming Assessment in Multicultural Europe (NAME) [28] and the switching verbal fluency test (TFA-93) [29], were employed more sporadically.

Among SCT, the most frequently used were the Animal Fluency Test (*n = *58) [30], Trail Making Test (TMT, *n = *48) [27], Rey–Osterrieth Complex Figure (ROCF, *n = *21) [28], and Stroop Test (*n = *23) [31].

Compared to patients assessed only with SCR, those who underwent NPS were older (68.2 ± 11.1 vs. 62.8 ± 14.5 years; p = 0.012) and had lower MoCA scores (in a subset of 58 individuals; 15.9 ± 5.0 vs. 21.4 ± 5.1; *p* < 0.001). There were no significant group differences in terms of education level, sex, knowledge of Italian, WBG-2, primary language, time frame, or final diagnostic classification (NON, SCD, MCI, DEM).

## Discussion

This study provides a longitudinal description of how clinical practice within an Italian CCDD has evolved over nearly a decade in response to the increasing CALD patients referrals.

A striking finding was the 180% increase over time in both the absolute number and proportion of CALD patients referred to the CCDD. This increase occurred in the absence of significant changes in age, sex, education, country income level, or linguistic proficiency, suggesting continuity in the sociodemographic profile of the referred population. In line with national demographic trends, population ageing and long-term migration dynamics likely contribute to the growing demand for cognitive assessment among migrants. However, consistent with data from the ImmiDem nationwide survey [9], which documented substantial regional variability in access to CCDDs, our single-centre findings should not be interpreted as evidence of an improvement in referral pathways at the national level. Rather, they reflect local service dynamics within a high-migration urban context.

The earlier exploratory study conducted in Rome in 2018 [7] and the retrospective analysis previously published by our group in 2019 [8] described CALD patients as a numerically limited but highly heterogeneous group, with dementia often diagnosed at advanced stages and assessments largely relying on standard cognitive tools. While CALD patients still represent a minority of referrals in the present cohort, we observed a shift in diagnostic distribution toward milder conditions, with a higher proportion of subjective cognitive decline and mild cognitive impairment and a relative reduction in dementia diagnoses, despite the mean MMSE score remained similar (23.4 ± 6.2 vs. 23.4 ± 6.3). This trend mirrors what has been observed in Italian patients attending CDCDs [32] and may reflect either earlier-stage referrals or improved diagnostic abilities, likely resulting from increased awareness and better access to culturally appropriate diagnostic tools.

From an European perspective, our findings complement those of the EADC surveys by providing a longitudinal, within-centre view of practice changes. While EADC studies highlighted persistent heterogeneity across centres in preparedness, tool availability, and use of interpreters, our data illustrate how a single memory clinic can progressively adapt its clinical approach over time.

The increasing use of cross-cultural screening tools represents one of the most relevant changes observed in our cohort. When RUDAS was compared with MMSE and MoCA, we found good overall concordance, in line with previous studies conducted in multicultural clinical settings. Importantly, RUDAS scores were not significantly associated with age or education, whereas MMSE and MoCA showed the expected demographic influences. This finding is consistent with existing literature supporting the relative robustness of RUDAS against sociodemographic bias and reinforces its suitability for CALD populations [33].

The introduction of CCTs represents another substantial evolution in practice. These tools were frequently administered alongside standard cognitive tests rather than replacing them, reflecting both the complementary diagnostic information they provide and the current limitations in the availability, normative data, and domain coverage of CCTs. The growing involvement of professional cultural mediators, especially during the second observation period, further underscores the complexity of cross-cultural assessment and aligns with recent European recommendations on interpreter-mediated neuropsychological evaluation [34].

The heterogeneity of languages, countries of origin, and educational backgrounds observed in our cohort mirrors that reported in previous Italian and European studies and continues to pose significant challenges for clinicians. These challenges include ensuring adequate cultural competence, selecting appropriate instruments (carefully adapted into target language [35]), managing interpreter-mediated assessments [34, 36], and interpreting test results in the absence of robust normative references [37]. In this context, our findings reinforce the notion that diagnostic accuracy in CALD patients depends not only on test selection but also on the broader clinical framework within which assessment is conducted.

This study has several limitations that need to be addressed. Foremost is its single-centre design, albeit in an experienced setting, and its retrospective nature spanning ten years, which introduces variability in data collection and a lack of uniformity in diagnostic procedures. However, in the absence of other comparable studies, we believe our findings offer valuable insights into evolving clinical practices and can inform future research and health policies. Additional limitations include the lack of precise information on Italian language communication skills and on acculturation, both critical aspects for future studies. Furthermore, the present analysis focused primarily on diagnostic staging, without detailed examination of specific neurocognitive profiles or underlying aetiologies.

Our study can serve as a trailblazer for further research, which should hopefully follow the ECLECTIC paradigm [38] which provides a comprehensive framework for investigating the multifaceted determinants of cognitive assessment in multicultural contexts. Specifically, future research should aim to:

- (E) Explore and clarify the effects of level and quality of EDUCATION, identifying reliable proxies for educational attainment in cross-cultural contexts [39, 40];
- (C) Raise awareness among healthcare professionals about patients' (and their families') background: history, customs, expectations, social roles and rules, religion and traditions, differences between cultures, views on cognition and cognitive impairment; measures and indicators for CULTURE should be part of this issue [41, 42];
- (L) Define the communicative competence in the host LANGUAGE, investigate the values ​​and biases of mediator-assisted cognitive assessment, and the reliability of mediators [43, 44];
- (E) Outline, following a health resource use model [45], the barriers and facilitators to accessing health services, highlighting factors that contribute to opinions about the health system and resource use; this area should include ECONOMIC issues such as availability and affordability [46, 47];
- (C): Examine how education, language, culture, and socioeconomic status interact to influence COMMUNICATION between patients and clinicians [48, 49];
- (T): Control for the TEST SITUATION, known to be mediated by many of the previous points (education, language, culture, economic issues), increasing awareness of limitations and biases both on the choice of tasks and on the means to overcome limitations (translation, adaptation or substitution) [35, 50];
- (I): Consider how cognitive potential is framed within different sociocultural models of INTELLIGENCE CONCEPTUALIZATION [51, 52];
- (C): Consider the CONTEXT OF IMMIGRATION (who, why, for how long, where, from where) as a crucial issue in the interpretation of patients' attitudes, responses and expectations; acculturation (integrated with the identification of its measures or proxies) and elements related to minorities (cultural, linguistic, ethnic) should be part of this research area [53, 54].

Many of these ECLECTIC components are interdependent, particularly education, language, culture, economic issues, and the test setting. Research and clinical practice must adopt a multidimensional approach that reflects this complexity.

## Conclusions

In the context of Italy’s ongoing demographic transformation, with an aging population and increasing cultural and linguistic diversity, dementia services must adapt to ensure equitable and effective care. This study highlights the progressive implementation of intercultural clinical practices within an Italian memory clinic, including the use of cross-cultural cognitive tools and professional mediators, which have contributed to more accurate and inclusive diagnostic processes.

The marked increase in referrals of CALD patients underscores both the growing need for culturally sensitive services and the enhanced accessibility of specialized memory care. Despite the persistence of sociodemographic patterns, diagnostic trends suggest earlier referrals and greater clinical awareness, likely supported by improved use of adapted assessment instruments.

Nevertheless, several challenges remain. These include limited availability of validated cross-cultural neuropsychological tools, variable training in cultural competence among clinicians and mediators, and persistent barriers to access and affordability of care. Further multicentre, prospective studies are needed to address these issues and to validate the observed trends.

## Data Availability

The data that support the findings of this study are available on request from the corresponding author.
